# Identification and Evaluation of Mobile Applications for Self-Management of Diet and Lifestyle for Patients with Inflammatory Bowel Disease

**DOI:** 10.1093/jcag/gwad029

**Published:** 2023-09-16

**Authors:** Stephanie L Gold, Brandon A Chiew, Vidya Rajagopalan, Celeste M Lavallee

**Affiliations:** Division of Gastroenterology, Icahn School of Medicine at Mount Sinai, Gustav Levy Place, New York, NY 10029, USA; Department of Medicine, Cumming School of Medicine, Health Sciences Centre, Foothills campus, University of Calgary, 3330 Hospital Drive NW, Calgary, Alberta T2N 4N1, Canada; Department of Medicine, Cumming School of Medicine, Health Sciences Centre, Foothills campus, University of Calgary, 3330 Hospital Drive NW, Calgary, Alberta T2N 4N1, Canada; Department of Medicine, Cumming School of Medicine, Health Sciences Centre, Foothills campus, University of Calgary, 3330 Hospital Drive NW, Calgary, Alberta T2N 4N1, Canada

**Keywords:** self-management, mobile applications, rating, MARS, inflammatory bowel disease

## Abstract

**Background:**

Mobile health applications (apps) providing diet and lifestyle self-management programs to patients with inflammatory bowel disease (IBD) are emerging. The objective of this study was to evaluate current apps available in the US and Canada based on app quality, perceived impact on diet and mental health and comprehensiveness to support self-management.

**Methods:**

The Apple iOS and Google Play app stores were searched for terms related to IBD. Apps were included if they targeted diet and lifestyle behaviours for patients living with IBD and were available to the general public. Apps were excluded if they were not specific to IBD, not available in English, did not target diet or lifestyle therapy, were not available in the US and Canada, or did not offer stand-alone self-management programs. The Mobile App Rating Scale was used to assess mobile app quality.

**Results:**

A total of 1,512 apps were identified through the app stores. Six apps met inclusion criteria. *My IBD Care: Crohn’s and Colitis* received the highest quality rating and *Lyfe*^*MD*^ received the highest overall app rating. Only these two apps provided behaviour tracking over time, and three (50 percent) apps provided good-quality information.

**Conclusions:**

While many IBD-related apps exist, few support self-management of diet and lifestyle behaviours. The My IBD Care and Lyfe^MD^ apps had the highest ratings and can be used to track lifestyle behaviours. The effectiveness of these apps to improve behaviours, and subsequently impact the disease course and quality of life, should be explored in future studies.

**Key messages**

- Use of mobile health applications can improve levels of self-management in adults with chronic disease.
- Despite evidence for self-management, the number of mobile health applications that support self-management of diet and lifestyle therapy for Canadian and US patients with inflammatory bowel disease (IBD) is quite limited.
- This study identified mobile health applications that offer diet and/or lifestyle therapy self-managements programs for Canadian and US patients with IBD, and rated them for quality, comprehensiveness, and perceived impact on diet and mental health to improve outcomes. The results of the study could be used to guide patients on the selection of a mobile health application to support self-management of their disease.

## Introduction

The prevalence of inflammatory bowel disease (IBD), including Crohn’s disease (CD) and ulcerative colitis (UC) is approximately 1.3 percent and 0.7 percent in the US and Canada, respectively.^1,2^ While the pathogenesis of IBD remains unclear, the rising incidence in western societies suggests that in a genetically susceptible person, environmental exposures, including diet and lifestyle factors, contribute to this chronic inflammatory state.^3–5^ Although the specific roles these lifestyle factors play in the inflammatory cascade remain unknown, there is evidence suggesting diet, physical activity, stress management, and sleep quality interventions can improve disease symptoms, gut inflammation, postoperative outcomes, and quality of life (QoL).^6–15^ Moreover, the recent STRIDE-II update on therapeutic targets in IBD noted that in addition to normalization of traditional markers of inflammation, such as C-reactive protein and calprotectin, and clinical markers of remission, physicians should also target improved QoL.^16^

While large, academic IBD centres may provide multidisciplinary support from dedicated nutritionists, psychologists, therapists, and pharmacists, many patients do not have access to these services and patients report a need for diet and lifestyle interventions. Close to 90 percent of patients with IBD regarded guidance on diet as important, yet less than 16 percent received information on diet to manage their disease.^17^ Likewise, 46.1 percent of patients with IBD felt that not enough emphasis was placed on the importance of physical activity by healthcare providers (HCPs) and 13.2 percent wanted more support to improve activity levels.^18^ Similarly, IBD is associated with a significant increase in psychological disorders, including anxiety and depression, yet few patients receive mental health supports.^19^

For individuals with limited access to multidisciplinary supports, mobile health (mHealth) apps that provide guidance on diet, physical activity, and mental health management strategies may fill this void. An increasing number of mHealth apps have emerged that offer self-management strategies to improve IBD symptoms and QoL.^20^ Therefore, the objective of this study was to evaluate IBD-focused (mHealth) apps available to patients in Canada and the US that support self-management of diet and lifestyle therapies for app quality, perceived impact on diet and mental health, and comprehensiveness of diet- and lifestyle-related features.

## Methods

### App selection

#### Search strategy

The Apple iOS App Store and Google Play store were searched by three reviewers (CML, VR, SLG) using the terms “IBD,” “inflammatory bowel disease,” “UC,” “colitis,” and “Crohn’s” in February 2022. Apps were included whether they were available in both stores or just one, and whether they were free or required payment to subscribe.

#### Eligibility criteria

Apps were included if they targeted diet and lifestyle therapies for patients living with IBD, or their caregiver(s), and were available to the general public. Apps were excluded if they were not relevant to IBD, not available in English, did not target diet or lifestyle therapies, were not available in both Canada and the US, included only virtual care without a stand-alone self-management option, or were not specific to IBD.

#### Screening and review

Apps were prescreened by reviewing their descriptions in the app store. Apps that appeared to meet the inclusion criteria according to their descriptions were downloaded and screened further for eligibility. Apps that met inclusion criteria after screening were evaluated by three reviewers (CML, VR, and SLG). All of the reviewers in this study (three individuals) underwent training to utilize the MARS prior to reviewing the mHealth apps. All three individuals reviewed the MARS and any component of the evaluation that was not clear to the raters was reviewed together to standardize responses prior to the review of the mHealth apps. When reviewers disagreed on a rating, they discussed their reasons for the rating. However, each reviewer still determined their rating autonomously after the discussion. Of note, the MARS tool includes a subjective assessment component, which includes questions on whether the reviewer would recommend this app to others, how often the reviewer would use this app, and whether the reviewer would pay to use the app. After review of the MARS tool, a decision was made to exclude the subjective quality assessment given the goal to review each app as objectively as possible and reduce the risk of bias among all three reviewers in the study.

### Assessment of quality, perceived impact, and relevant app features

#### Quality

The Mobile App Rating Scale (MARS) is an objective, validated tool to assess mobile app quality.^21^ The MARS previously demonstrated excellent internal consistency (Cronbach’s alpha = 0.90) and interrater reliability (intraclass correlation coefficient [ICC] = 0.79).^21^ The tool consists of four objective quality subscales with a total of 19 items (engagement = 5 items, functionality = 4 items, aesthetics = 3 items, and information = 7 items) and a subjective quality subscale with a total of four items scored separately.^21^ Items present within the subscales are rated on a five-point Likert scale: 1 = inadequate, 2 = poor, 3 = acceptable, 4 = good, and 5 = excellent. Any item within a subscale that is not present in the app was rated as “not available” and was not included in the calculation to determine the mean score for that subscale. For the purposes of this study, the four objective quality ratings of the MARS were used to assess app quality, with one adaption. Item 14 of the MARS rates the quality of user goals included within the app. Given that goal setting is an important aspect of behaviour change,^22^ if no goal-setting activities were available in the app, the app received a rating of 1 (inadequate) rather than “not available.” The subjective quality subscale was not used in this study.

#### Perceived impact on diet and mental health behaviours

The six-item app-specific subscale of the MARS was adapted to evaluate the perceived impact of the app on the user’s awareness, knowledge, attitudes, intention to change, support-seeking behaviour, and likelihood of behaviour change for both diet and mental health to improve IBD outcomes. Items in the app-specific subscales were rated on a five-point Likert scale: 1 = strongly disagree, 2 = disagree, 3 = neither agree or disagree, 4 = agree, and 5 = strongly agree. The adapted questions and descriptors used are shown in Table 1. The app-specific item that rates the likelihood the app will increase the user’s intentions or motivation to change was adapted to instead assess availability of tools to support behaviour change related to diet and mental health. The help-seeking item was adapted to assess whether the app either encouraged or provided social or professional support. The behaviour change score was based on the scores of the other items within the subscale.

**Table 1.** App-specific items and descriptors to assess perceived impact of diet and mental health behaviours on IBD.

Rating	Adapted question	Strongly disagree	Disagree	Neutral	Agree	Strongly agree
Item		1	2	3	4	5
Awareness (about the condition)	This app is likely to increase awareness of the importance of addressing diet/mental health to improve IBD outcomes	No information or tracking present	Information or tracking but not both	General information or comments, but not specific to the user	Collated information based on their tracking	In-depth information or user-specific feedback based on their tracking
Knowledge (about diet and IBD or about mental health and IBD)	This app is likely to increase knowledge/understanding of diet/mental health to improve IBD outcomes	No information present	Blog post or input from other users based on their experiences	Articles	General courses	Articles and interactive/personalized courses
Attitude (reinforcement for taking steps toward positive behaviour change)	This app is likely to attitudes toward improving diet/mental health to improve IBD outcomes	No reinforcement	Offers recognition for tracking or achieving goal, but no reward	Offers a reward for tracking	Offers a reward for achieving goals	Offers a reward for tracking and achieving goals
Programs of support related to diet	This app is likely to increase awareness of the importance of addressing diet/mental health to improve IBD outcomes	No programs available	Offers meal plans or recipes but not both	Offers meal plans and recipes	Offers motivational interviewing or other behaviour change program	Offers motivational interviewing or other behaviour change program along with meal plans and recipes
** **Programs of support related to mental health	This app is offers tools to support behaviour change related diet/mental health to improve IBD outcomes	No tools available	Includes noninteractive tools to help mental health (e.g., information on tools to help stress, breathing, mindfulness)	Includes interactive yoga, breathing, mindfulness activities (such as video or audio)	Offers CBT but no other programs	Offers CBT along with yoga, breathing, and mindfulness activities
Encouragement to seek social or professional support	This app is likely to encourage seeking further social or professional support for diet/mental health to improve IBD outcomes	No guidance on seeking help	Encourages connection with community or with HCP (e.g., appointment tracking/reminders)	Provides access to a coach or other nonHCP	Recommends consulting an RD/psychologist/MD	Provides an opportunity to book a consult with an RD/psychologist/MD
Behaviour change	This app is likely to increase positive behaviours around diet/mental health to improve IBD outcomes	1–5 points from above	6–10 points from above	11–15 points from above	16–20 points from above	21–25 points from above

Abbreviations: CBT, cognitive behavioural therapy; HCP, healthcare provider; MD, medical doctor; RD, registered dietitian.

#### Comprehensiveness of relevant app features

Each app was evaluated for inclusion of eight features that could be used to build comprehension. These app features were chosen by an expert consensus utilizing data from the literature demonstrating the importance of goal setting and behavioural change in patients with IBD.^23^ Apps were rated for their inclusion of the following features: (1) information or support for diet; (2) information or support for physical activity; (3) information or support for mental health; (4) ability for the user to track diet, physical activity, or mental health-related behaviours; (5) use of interactive educational activities to promote user engagement (e.g., instructional videos or questions to answer to reinforce learning after completing a course); (6) live in-app interaction with other patients, a coach, or an HCP; (7) ability to choose and track goals based on current symptoms or lifestyle behaviours; and (8) inclusion of behaviour-change supports (i.e., cognitive behavioural therapy [CBT] or components of motivational interviewing). Apps were given one point for each feature present in the app, for a total comprehensiveness score between zero and eight. This score was converted into a score out of five to ensure equal weighting of the comprehensiveness score in the calculation of the overall mean app score.

### Data analysis

Descriptive statistics analyses were conducted. The mean scores for each of the four quality subscales of the MARS (engagement, functionality, aesthetic, and information), the perceived impact on each diet and mental health to improve IBD outcomes, and the comprehensiveness of relevant app features were calculated. The overall mean score for each app was calculated by averaging quality, comprehensiveness, and perceived impact on diet and mental health to improve IBD outcomes. To examine inter-rater reliability, ICCs using a two-way mixed model with absolute agreement for a single measure were used. To interpret the ICC, Fleiss criteria were used where ICCs >0.75 are excellent.^24^

## Results

### Identified apps

**Figure 1.**
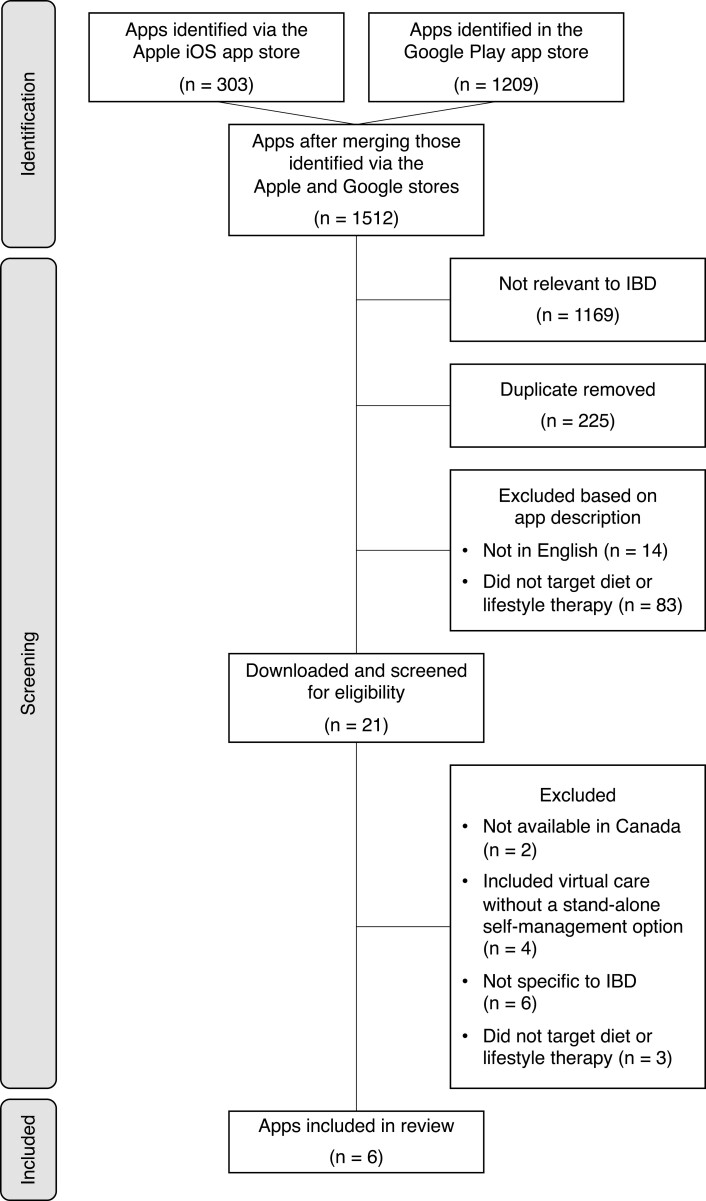
The medical app identification and screening process is outlined here. Ultimately, 6 apps were included and the remainder were excluded for the reasons highlighted in the figure.

Out of 1,512 apps identified in the Apple iOS app store and Google Play store, 21 apps were downloaded for a more detailed screening of eligibility (fig. 1). Four were downloaded for further screening were used to provide virtual care for IBD led by an individual HCP or a multidisciplinary team, such as gastroenterologists, nurses, dietitians, social workers, and behavioural specialists, in conjunction with the app. Although these apps provided support for diet or lifestyle therapy, they were excluded from the assessment because they did not offer a stand-alone self-management option users could use without also signing up for HCP-led virtual care. The six apps not specific to IBD included education or support for irritable bowel syndrome and/or digestive disorders in general, in a manner that the user would not be able to distinguish guidance specific to IBD from guidance specific to irritable bowel syndrome or other digestive disorders. Of the three apps that were excluded because they did not offer education or support for diet or lifestyle, two included food, symptom or medication trackers, and one was used to alert the user’s physician of a potential disease flare. Features of IBD apps that did not offer education or support for diet or lifestyle therapy are shown in Table 2.

**Table 2.** Features of IBD apps that did not offer education or support for diet or lifestyle therapy were excluded in the initial screening.

Features	Number of apps
Food, symptom, or bowel movement trackers without an education or a lifestyle program	30
Community or social support structure trackers without an education or a lifestyle program	3
Education on IBD but in areas unrelated to diet or lifestyle therapy	21
Education targeted toward healthcare professionals rather than patients	14
Not available to the general public because they were developed specifically for research projects	4
Not available to the general public because they were developed for patients at a particular hospital	1
Used in conjunction with at-home faecal calprotectin test kits	3
Used for attendees at IBD-related conferences	2
Help users locate nearby washrooms	2
Used to find a physician	1
Used as a fundraising platform	1
Provided a virtual reality experience for people without IBD to better understand what it is like to live with the disease	1

IBD, inflammatory bowel disease.

### App ratings

Ratings for perceived impact, comprehensiveness, MARS app quality, and overall app scores are shown in Tables 3–6, respectively. *Lyfe*^*MD*^ received the highest overall app score (15.5/20.0), based on an average of the scores for app quality, perceived impact on diet and on mental health, and comprehensiveness. *Lyfe*^*MD*^ scored the third highest on the MARS quality rating, with a score corresponding to “acceptable,” and scored highest on comprehensiveness. The *Lyfe*^*MD*^ app received the highest score for perceived impact on diet, and second highest for perceived impact on mental health. Of note, the Lyfe^MD^ app is available free of charge to all patients with IBD in Canada for 12 months (by obtaining an access code from the company). For users outside of Canada, there is a fee to use the app.

**Table 3.** Ratings of perceived impact on diet (A) and mental health (B) to improve IBD outcomes based on the app-specific ratings of the MARS.^21^

App name	Awareness	Knowledge	Attitudes	Tools or programs of support	Help seeking	Behaviour change	App-specific Mean Score (DIET)
A) Perceived impact on diet							
Lyfe^MD^	5.0	3.0	2.0	5.0	3.0	4.0	3.7
My IBD care: Crohn’s and Colitis	1.0	1.0	1.0	1.0	1.0	1.0	1.0
MyGut	3.0	3.0	1.0	1.0	2.0	2.0	2.0
Colitis diary^a^	4.0	1.0	2.0	1.0	2.0	2.0	2.0
Crohn’s diary^a^	4.0	1.0	2.0	1.0	2.0	2.0	2.0
IBD fighter	4.0	1.0	1.0	1.0	1.0	2.0	1.7

App name	Awareness	Knowledge	Attitudes	Tools or programs of support	Help seeking	Behaviour change	App-specific mean score (mental health)
B) Perceived impact on mental health							
Lyfe^MD^	5.0	5.0	2.0	5.0	3.0	4.0	4.0
My IBD care: Crohn’s and Colitis	5.0	5.0	2.0	4.3	4.0	5.0	4.2
MyGut	3.0	3.0	1.0	2.0	2.0	2.0	2.2
Colitis diary^a^	4.0	1.0	2.0	1.0	2.0	2.0	2.0
Crohn’s diary^a^	4.0	1.0	2.0	1.0	2.0	2.0	2.0
IBD fighter	4.0	1.0	1.0	1.0	1.0	2.0	1.7

^a^Used the same platform by the same developer, but with different titles.

**Table 4.** Ratings of comprehensiveness based on the inclusion of relevant app features. These app features were chosen by an expert consensus utilizing data from the literature demonstrating the importance of goal setting and behavioural change in patients with IBD.^23^

App feature	Lyfe^MD^	My IBD care: Crohn’s and Colitis	MyGut	Colitis diary	Crohn’s diary	IBD fighter
Diet addressed	1	0	1	1	1	1
Physical activity addressed	1	1	1	1	1	1
Mental health addressed	1	1	1	1	1	1
Ability to track behaviours	1	1	1	1	1	1
Interactive education to promote user engagement	1	1	0	0	0	0
Live in-app interaction with other patients, a coach, or a health professional	0	0	0	0	0	0
Ability to choose goals or tracking specific to the user	1	1	0	0	0	0
Behaviour change program	1	0.5	0	0	0	0
Total	7	5.5	4	4	4	4
Comprehensiveness score out of 5	4.4	3.4	2.5	2.5	2.5	2.5

IBD, inflammatory bowel disease.

**Table 5.** Ratings of app quality based on the engagement, functionality, aesthetics, and information quality subscales of the MARS.^21^

App name	Version	Developer	Cost (Canadian $)	Engagement	Functionality	Aesthetics	Information quality	Mean MARS quality score
Lyfe^MD^	1.2	Lyfe^MD^ Inc.	No charge	3.2	3.0	3.7	3.9	3.4
My IBD care: Crohn’s and Colitis	4.1.1	Ampersand Health Limited	No charge	3.9	3.9	4.3	4.8	4.2
MyGut	1.1.2	Crohn’s and Colitis Canada	No charge	2.6	3.6	4.5	3.8	3.6
Colitis Diary^a^	1.7.98	cellHigh	6.99	2.7	3.4	2.7	3.0	2.9
Crohn’s Diary^a^	1.7.98	cellHigh	6.99	2.7	3.4	2.7	3.0	2.9
IBD fighter	1.2.3	WoMoApp., s.r.o.	No charge	1.8	4.1	3.3	2.6	3.0

Abbreviations: MARS, mobile application rating scale.

^a^Used the same platform by the same developer, but with different titles.

**Table 6.** Overall app scores and ratings of quality, perceived impact on diet and mental health to improve IBD outcomes, and comprehensiveness of relevant app features.

App name	MARS quality score	Perceived impact on diet to improve IBD outcomes	Perceived impact on mental health to improve IBD outcomes	Comprehensiveness of relevant app features	Overall app score (Total sum)
Lyfe^MD^	3.4	3.7	4.0	4.4	15.5
My IBD care: Crohn’s and Colitis	4.2	1.0	4.2	3.4	12.8
MyGut	3.6	1.8	1.9	2.5	9.8
Colitis diary^a^	2.9	1.8	1.8	2.5	9.0
Crohn’s diary^a^	2.9	1.8	1.8	2.5	9.0
IBD fighter	3.0	1.7	1.7	2.5	8.9

^a^Used the same platform by the same developer, but with different titles.

Abbreviations: IBD, inflammatory bowel disease; MARS, mobile application rating scale.

*My IBD Care: Crohn’s and Colitis* received the second highest overall app score (12.8/20.0) and the highest MARS quality rating, which corresponded with “good.” *My IBD Care: Crohn’s and Colitis* scored highest on perceived impact on mental health, however, it did not address diet and therefore it received a lower comprehensiveness score and the lowest score for perceived impact on diet to improve IBD outcomes.

*MyGut* ranked third for overall app score (9.8/20.0) and received the second highest MARS quality rating, which corresponded with “acceptable,” scored low in its perceived impact on both diet and mental health and on comprehensiveness of relevant app features.

*Colitis Diary* and *Crohn’s Diary* received identical ratings, leaving them with the fourth highest overall app score (9.0/20.0). While these apps have different names, they are from the same developer and use the same platform, hence the identical scores for all parameters. The MARS quality ratings for these apps were the lowest and were considered poor.

*IBD Fighter* received the lowest overall app score (8.9/20.0) and received a MARS quality rating considered acceptable. The overall score for *IBD Fighter* was low due to its perceived impact on diet and on mental health to improve outcomes.

Only *Lyfe*^*MD*^ and *My IBD Care* supported patients in tracking behaviours of their choosing and/or based on their current symptoms or lifestyle (Table 4). Based on MARS item 15, quality of information (a sub-item of overall information quality), *Lyfe*^*MD*^ scored 4.7, *My IBD* Care scored 4.3, and *MyGut* scored 4.3. Quality was considered good if it was relevant, appropriate, coherent, and correct in relation to the purpose of the app. The remaining three apps received acceptable quality of information scores (2.7–3.0) on MARS item 15 for app content that was moderately relevant, appropriate, or coherent and that appeared correct.

## Inter-rater reliability

For all four scales (overall MARS score, app comprehensiveness, focus on diet, and focus on mental health) the ICCs indicated excellent agreement (MARS ICC = 0.75, 95 percent CI = (0.35–0.96; Comprehensiveness ICC = 0.97, 95 percent CI (0.88–0.99); Diet ICC =1.0; Mental health ICC (3,1) = 0.99, 95 percent CI = 0.98–0.99).

## Discussion

This study identified and evaluated the quality of Canadian and US mHealth apps designed for diet and lifestyle self-management for patients with IBD. After an extensive search of the available mHealth apps, only six IBD-focused apps for lifestyle modification were identified. Based on a validated, published app-scoring system, three of the identified apps provided good-quality information. The two apps with the highest overall rating were *Lyfe*^*MD*^ and *My IBD Care: Crohn’s and Colitis.* These two apps differentiated from the others because they provided interactive education and the ability for the user to choose their lifestyle goals and behaviours to modify or track. *Lyfe*^*MD*^ offered the most comprehensive support because it provided programs for diet, physical activity, and mental health self-management. Of note, the Lyfe^MD^ app is available free of charge to all patients with IBD in Canada for 12 months (by obtaining an access code from the company). For users outside of Canada, there is a fee to use the app.

*My IBD Care: Crohn’s and Colitis* was rated as a high-quality mHealth app to support self-management of mental health; however, it was less comprehensive than *Lyfe*^*MD*^ because it did not offer diet therapy. Our findings were supported by a recent study evaluating the quality of commercially available IBD-related apps,^25^ which similarly used the MARS to rate the overall quality of the reviewed mHealth apps. That study identified very similar overall quality scores for the *My IBD Care: Crohn’s and Colitis* app (overall quality rating 4.62) as well as the *IBD Fighter* app (overall quality rating 3.36), supporting our findings in the current study, although the *Lyfe*^*MD*^ app was not reviewed in that recent manuscript.

Lifestyle modification programs have been suggested for patients with IBD to improve their physical and mental health, in addition to reducing symptoms from the underlying disease. Diet is now an established lifestyle therapy for patients with IBD with evidence demonstrating improved remission with dietary therapy.^10,11,13,14,26^ Physical activity has been associated with increased bone mineral density,^6^ longer rates of remission,^8^ and improved fatigue^15^ and QoL^9,15^ in patients with IBD. nonpharmacological treatments such as CBT and mindfulness techniques may be beneficial for patients with IBD.^27^ Mindfulness activities including meditation and relaxation significantly improved IBD symptoms, QoL, and inflammatory biomarkers,^7^ and practicing yoga was shown to reduce perceived stress, which in turn was associated with reduced disease activity and increased QoL.^12^ Online programs demonstrate similar results. An online stress-reduction intervention in patients with IBD improved perceived stress, mental health, and QoL, though it did not impact IBD symptoms or inflammatory biomarkers,^28^ while an online yoga program effectively reduced symptoms of irritable bowel syndrome.^29^ Furthermore, virtual, guided cognitive behaviour therapy is an effective treatment for anxiety and depressive disorders in patients with IBD^30^; While many mental health treatments require an intervention led by a provider, yoga, relaxation techniques such as breathing, and meditation can be practiced by the patient on their own. Self-guided CBT in patients with IBD has not been studied, but positive results from a trial in patients with irritable bowel syndrome^31^ indicate that CBT could be practiced by the patient on their own. While this evidence supports the use of lifestyle modification programs, it is imperative that mHealth apps provide evidence-based guidance to patients. *Lyfe*^*MD*^, *My IBD Care: Crohn’s and Colitis* and *MyGut* were the only apps that provided evidence-based information. *Lyfe*^*MD*^ was the only app that offered a practical implementation strategy to incorporate guideline-based recommendations for diet. *Lyfe*^*MD*^ and *My IBD Care: Crohn’s and Colitis* were the only apps that offered evidence-informed behaviour-change programs along with activities related to mental health, such as yoga, breathing, and mindfulness.

Studies have demonstrated that a multidisciplinary approach to caring for patients results in improved QoL, however, the impact of lifestyle modification on disease course remains unclear.^32,33^ Recently, the International Organization for the Study of Inflammatory Bowel Diseases published a consensus statement on lifestyle, behaviour, and environmental modification recommendations for patients with IBD.^23^ The committee recommended lifestyle modifications such as smoking cessation, use of diet therapies that are supported by evidence and involve monitoring for improvement in inflammation and potential nutritional deficiencies, regular physical activity as tolerated, and routine screening for mental health disorders. The authors note that as with pharmacotherapy, the effectiveness of these lifestyle and behaviour modifications relies on adherence, which they suggest is a collective responsibility between the patient and their HCP.^23^ The literature on adherence to various lifestyle modifications in patients with IBD is quite poor and limited to a small number of smoking cessation and diet studies.

While many multidisciplinary centres offer access to nutrition and psychiatric care, these services are not universally available, leaving many patients without access. Given this gap and recent advances in smart phone technology, many physicians are looking at mHealth technology to increase access to guide patients who may not otherwise be able to access this type of care.^34^ Across medical disciplines, the use of mHealth apps has increased exponentially in the past few years; in a recent survey of over 2,000 adults with diabetes, hypertension, heart disease, or lung disease, roughly 60 percent were utilizing mHealth apps to communicate with their HCP, access their medical record, or make decisions for treating an illness or condition.^35^ The use of mHealth apps has also increased in IBD; in 2016 Con et al. reviewed 26 IBD-focused mHealth apps that offered diet and mood diaries, IBD symptom trackers, community support, or disease-related information.^36^ The researchers concluded that these novel tools may prove useful as an adjunct to traditional care in patients with IBD.^36^ To this point, in studying the HealthPROMISE app, Khan et al. demonstrated that patient engagement in mHealth apps was highest when there was significant physician involvement and feedback.^37^

While mHealth apps show promise as tools to support patient self-management, limited professional involvement,^36,37^ recommendations that do not consistently align with medical guidelines,^36^ and the significant time and cost to developing and maintaining these tools may limit their usability and sustainability. Furthermore, respondent fatigue and technical problems are seen by patients as barriers to using these apps.^38^ Thus, well-designed mHealth apps should offer good-quality information and engaging content with few technical problems. This study demonstrated that there are only a few of such mHealth apps available in Canada and the US to help patients self-manage their IBD.

There are a few limitations to this study. First, only apps that were available in English were included; inclusion of apps in multiple languages would provide a more thorough review of the available mHealth apps to the worldwide population with IBD. Moreover, we only evaluated apps that were available in both Canada and the US. Another potential limitation is that two reviewers of the apps provide contracted services to Lyfe^MD^; however, the MARS tool reduces the likelihood of bias because it provides a clear description for the requirements that must be met for each item for an app to be given a certain score. Furthermore, a third, unbiased reviewer with no connection to Lyfe^MD^ Inc. (SLG) was included to further reduce any bias. In fact, between all three reviewers, the ICCs were all very strong, suggesting that there was limited bias in the evaluation of these apps. Although the MARS is a validated app assessment tool, this tool certainly has inherent limitations. The quality of characteristics such as such as engagement, functionality, and aesthetics is not always synonymous with the quality of the content within the app or the program(s) that it offers. For example, the accuracy of the app as evaluated by the MARS relates to the accuracy of app description in the app store rather than the accuracy of the information provided within the app itself. Moreover, despite the benefits of goal setting on behaviour change,^22^ if goals are not available within the app, the MARS tool simply eliminates that item from the rating calculation rather than giving it a rating of zero (0). Finally, while the MARS can assess the perceived impact of the app on various target health behaviours, the tool does not assess the actual impact, or effectiveness, of the app on those behaviours.

Despite these limitations, this study is, to the best of our knowledge, the first to assess the availability and quality of Canadian and US mHealth apps supporting self-management of lifestyle behaviours and diet for IBD patients. Of the six identified mHealth apps, *Lyfe*^*MD*^ and *My IBD Care: Crohn’s and Colitis* apps had the highest overall rating. While these apps are likely helpful for all patients with IBD, they may be most beneficial for patients who do not have access to a multidisciplinary care team of practitioners trained in IBD to provide one-on-one guidance on diet and lifestyle therapies. Going forward, studies are needed to better understand the ability of these apps to improve diet and lifestyle behaviours and subsequent clinical outcomes, including QoL and disease activity in patients with IBD.

## Supplementary Material

gwad029_suppl_Supplementary_Checklist

## Data Availability

The data underlying this article will be shared upon reasonable request to the corresponding author.
